# *“How to prepare for end of life”:* Co-development of a community-based advance care planning intervention through participatory design

**DOI:** 10.1016/j.pecinn.2026.100489

**Published:** 2026-07-10

**Authors:** Sabrina Westergaard Jensen, Morten Rune Blichfeldt-Eckhardt, Helene Korvenius Nedergaard, Mette Aaby Smith, Hanne Irene Jensen

**Affiliations:** a Department of Anesthesiology and Intensive Care, Vejle, a part of Lillebaelt Hospital, University Hospital of Southern Denmark, Vejle, Denmark; b Department of Anesthesiology and Intensive Care, Kolding, a part of Lillebaelt Hospital, University Hospital of Southern Denmark, Kolding, Denmark; c Department of Regional Health Research, University of Southern Denmark, Odense, Denmark

**Keywords:** Advance care planning, Co-design, Participatory design, Health communication, Older adults, Intervention, End of life

## Abstract

**Objective:**

In this participatory design study, we investigated which inputs a workshop panel had for the content and format of an oral presentation about how to prepare for end of life, and which inputs they had for questions in two evaluation tools (questionnaires and an interview guide).

**Methods:**

We co-developed an intervention consisting of an oral presentation and evaluation tools. Two structured workshops were conducted with a workshop panel. The development followed three phases of a participatory framework: needs assessment, idea generation, and testing/retesting. The data included audio recordings, post-it notes and participant feedback. Post-it notes were thematically analyzed, inspired by affinity diagramming (the KJ Method), and feedback was analyzed by content analysis.

**Results:**

Participants identified key themes, including legal rights and ethical dilemmas related to treatment decisions, care options and pathways, psychosocial concerns regarding quality of life, clear communication with healthcare professionals, and conceptual clarity. The workshop panel suggested a narrative communication strategy to foster personal reflection and emotional resonance. Participants also recommended developing handouts to encourage further dialogue and highlighted the value of follow-up opportunities and support networks. Feedback on the presentation was on slide design, content clarity, and performance.

**Conclusion:**

This study identifies themes and formats that can guide the development of early advance care planning interventions perceived as meaningful and accessible. The findings also highlight which domains older adults consider important to address early.

**Innovation:**

This study is innovative in using a participatory co-development process with a workshop panel of older adults, relatives, and healthcare professionals to develop an early advance care planning intervention.

## Highlights

• An early ACP intervention was co-developed with a panel by a participatory design.
• Data consisted of post-its and feedback.
• Main themes included legal, ethical, medical, psychosocial, and concept clarity.
• A narrative approach was emphasized to support personal reflection and resonance.
• The intervention was pilot tested and adjusted for clarity and relevance.

## 1 Introduction

### 1.1 Background

Advance care planning (ACP) is defined by a multidisciplinary Delphi panel as “a process that supports adults at any age or stage of health in understanding and sharing their personal values, life goals, and preferences regarding future medical care.” [1]. In recent years, the field of ACP has evolved from a focus on the determination of the level of treatment and the completion of advance directives to a focus on communication and on preparing patients and surrogate decision-makers for future medical decision-making [2]. A recent systematic meta-review suggests that ACP interventions are associated with decreased hospital utilization in line with patients' preferences and with an increase in patients documenting their preferences, while evidence on patient quality of life and healthcare use, and goal-concordant care is inconsistent [3]. However, early discussions about ACP may ease the emotional burden [4] and reduce psychological distress among surrogate decision-makers, suggesting that ACP may achieve a central patient-identified goal of reducing the decision-making burden on relatives [2]. Despite these potential benefits, there seem to be significant barriers to ACP implementation [5]. Two reviews and a cohort study indicate that ACP is initiated relatively late in illness trajectories, and in cases where major treatment decisions have already been made [6], [7], [8]. In such cases, patients may lack the information and support to make choices that align with their values and preferences.

A Danish survey indicates a positive attitude toward ACP among older Danes (94%). However, almost half had not documented their end-of-life preferences [9], indicating barriers. Among older adults (≥65 years), awareness and knowledge of ACP and palliative care remain limited [10], [11], emphasizing the need for population-wide interventions. A scoping review of high-quality randomized trials found that more than 70% of intervention studies using patient-directed written materials, multimedia resources, and facilitated discussions reported beneficial effects [2], [12]. A digital intervention, PREPARE *for Your Care,* developed for older adults and their surrogates, has shown a significant increase in ACP documentation in medical records [13], [14], [15], greater patient empowerment to initiate ACP discussions during primary care visits, and an increase in goal-concordant care [16], [17], [18]. A systematic review emphasized the value of interactive and participatory approaches over passive information provision [19], and a cross-sectional study further indicated that knowledge alone rarely leads to decision-making, as psychological and emotional factors also influence individuals' readiness to engage in health behaviors [20].

Several previous studies conceptualize ACP as a set of health behaviors, enabling the identification of distinct stages of readiness among older adults to engage in ACP-related activities [21], [22], [23], [24]. Conceptualizing ACP within a health behavior framework can illustrate the complexity of older adults' engagement and can inform the design of interventions. For example, a participatory design protocol illustrates how behavior change theory can be integrated with co-design to develop an educational ACP intervention through workshops [25]. Complementing this, an intervention development study indicates that workshops can serve as an effective method for co-designing ACP interventions [26].

Involving end-users in the co-design of health interventions is increasingly recognized as essential to ensure their relevance, feasibility, and acceptability in real-world settings [27]. Especially in sensitive topics such as EOL care, user perspectives can provide valuable insights into how content, tone, and format should be shaped to resonate with the target population [28]. A participatory design study involving a 13-member Community Committee demonstrated the potential of community engagement in developing community-based ACP events [29]. The overall ACP readiness pre-to-post-event increased (*P* = 0.05), and readiness to document wishes increased significantly (*P* = 0.003) [29].

As outlined above, it is important to develop early ACP interventions that address both the informational and reflective needs of older adults. To ensure that such interventions are relevant, feasible, and resonate with the target audience, it is essential to integrate user perspectives into the design process.

### 1.2 Objective

In this participatory design study, we investigated which inputs and feedback a workshop panel had for the content and format of an oral presentation about how to prepare for end of life, and which inputs and feedback they had for questions in two evaluation tools (a questionnaire and an interview guide).

## 2 Methods

**Fig. 1:**
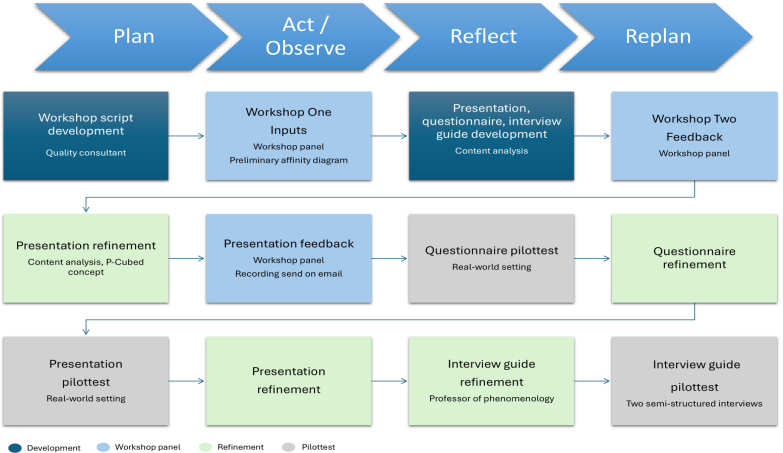
Flow diagram: The iterative processes of development, data collection, refinement, and pilottests, inspired by the action research spiral.

This article adheres to the Standards for Reporting Qualitative Research (SRQR) [30]. The iterative development processes were inspired by the action research spiral “plan, act, observe, and reflect” [31] (see Fig. 1 for a linear presentation of the iterative processes).

### 2.1 Study design

With this qualitative participatory design (PD) study, we co-developed an ACP presentation and evaluation tools in collaboration with a workshop panel consisting of patients, relatives, citizens, and healthcare professionals through two workshops.

### 2.2 Workshops

Workshops are a structured setting for creative group activities, idea generation, development, and problem-solving related to a specific issue [32]. Workshops in research incorporate participatory methodologies, emphasizing that collaborative engagement in co-creation activities facilitates deeper insights into the field and enhances the richness of the data [33].

#### 2.2.1 Framework

The workshops were guided by the first three phases of a four-phase PD framework designed to support meaningful and structured involvement of users in developing health interventions [34], [35], [36], [37] (see Theoretical Framework for the Workshops in Appendix 1). The framework consists of the following phases: 1) needs assessment, 2) idea generation, 3) testing and retesting, and 4) evaluation. These phases served as a guiding structure throughout the project. *Needs assessment* phase included the development of the project protocol and initial exploration of relevant themes during the first workshop. This was followed by the *idea generation* phase, also addressed in the first workshop, and the *testing and retesting* phase, which took place during the second workshop and a subsequent pilot test (see the appendix, Theoretical Framework for the Workshops). The final phase, *evaluation*, will be described later.

### 2.3 Participants and recruitment

Participants for the workshop panel were recruited by purposive sampling [38] to ensure representation of patients, citizens, relatives, and healthcare professionals. Three participants were recruited via the last author's professional network, and two of them had contributed to the development of the project during its initial stages. Patients and relatives were recruited through the patient and family advisory councils at the University Hospital of Southern Denmark. The size of the workshop panel was not predetermined. Participants were included based on their interest in the research topic, motivation to contribute, and were able to attend the workshops. The panel included three patients, two relatives, one citizen, and four healthcare professionals (see Table 1). One of the patients only attended the first workshop.

**Table 1:** Characteristics of the workshop panel.

Characteristics	Patients (*n* = 3)	Health care professionals⁎ (*n* = 4)	Relatives/Citizen (n = 3)	Total (*n* = 10)
Sex, n (%)				
Female	2 (66.7%)	2 (50.0%)	3 (100%)	7 (70%)
Male	1 (33.3%)	2 (50.0%)	0 (0%)	3 (30%)
Age (years)				
Median	57,0	56,5	68	63
Range	46–68	48–63	64–73	46–73
Missing⁎⁎	1	0	0	1

⁎ Health care professionals included a home care nurse, a hospital consultant specialized in anesthesiology and intensive care, a general practitioner, and a professor in end-of-life care.

⁎⁎ One participant in the patient group had missing age data and was excluded from age calculations.

### 2.4 Researcher reflexivity

The research team recognizes that existing relationships between the last author and three participants may have influenced both recruitment and the participants' motivation to engage in the study. Specifically, two participants had contributed to the development of the study protocol, and one had previously worked in the same department as the last author. Both the last and second authors served as moderators in two of three workshop groups and contributed to the development of the data collection approach. To minimize bias, the groups were deliberately structured to ensure that the last author did not moderate the session involving participants with whom she had a prior professional relationship.

### 2.5 Ethics

According to Danish law, research workshops do not need ethical approval. Before participation, all individuals received written and oral information about the purpose and procedures of the study. Participants were informed that parts of the workshops would be audio-recorded, that participation was entirely voluntary, and that they could withdraw from the study at any time. Written informed consent was obtained from all participants in accordance with the Declaration of Helsinki [39].

### 2.6 Setting

Two workshops were held (September 2nd and October 30th, 2024). The purpose was to co-develop the presentation content and accompanying evaluation tools, including questionnaires and an interview guide with a workshop panel. A quality consultant with expertise in workshop facilitation supported the planning to ensure meaningful involvement. A detailed script outlined roles, content flow, and materials (see Script for Workshop One in Appendix 1). The oral presentation, which constitutes the intervention, was titled *“Quality of Life at the End of Life: How to Prepare”.*

### 2.7 Workshop one – needs assessment/idea generation (three hours)

**Fig. 2:**
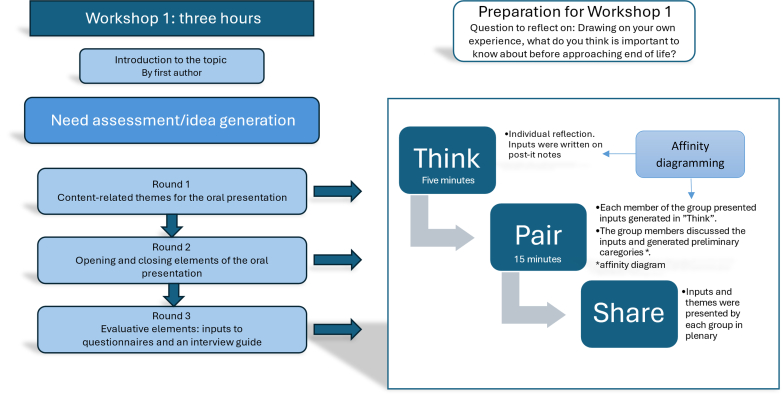
Activities of workshop one.

Prior to the first workshop, the panel members were asked by email to reflect on the following question as preparation for the workshop: “*Drawing on your own experience, what do you think is important to know about before approaching end of life*?”. The workshop (See Fig. 2) was introduced with an oral presentation of the topic by the first author, followed by a personal presentation from each panel member. Participants were divided into three mixed groups to create a smaller environment and encourage engagement from all members before sharing in plenum [33].

The workshop was structured by three rounds of a Think-Pair-Share process (see Fig. 2), which allowed for individual reflection and group-based ideation on potential themes, formats, and questions [40]. The purpose of each round was to generate inputs of content-related themes (round 1), opening and closing elements (round 2), and evaluative elements (questionnaires and interview guide) (round 3). Each round included:

• ‘Think’ (five minutes): Individual reflection, where each member wrote inputs on post-it's.
• ‘Pair’ (15 min): Group brainstorming, where each member presented the inputs generated by ‘Think’ and discussed all the inputs of the group, sorted the inputs in categories in a preliminary affinity diagram.
• ‘Share’: Each group presented essential inputs and discussions in plenary.

### 2.8 Development between workshops

The oral presentation was shaped by inputs from Workshop One and informed by a broad range of sources, including scientific literature, public discourse, opinion pieces, newspaper articles, and relevant non-fiction [41].

The questionnaires were developed based on insights from participant inputs from Workshop One, and further inspired by pre-existing survey instruments used in related research [42].

The interview guide was similarly informed by a few inputs from Workshop One, and most importantly, inspired by a qualitative interview study on EOL care (not yet published). The aim was to ensure that the guide would support open, meaningful dialogue while aligning with the themes raised in the presentation.

### 2.9 Workshop two – testing and feedback (two hours)

**Fig. 3:**
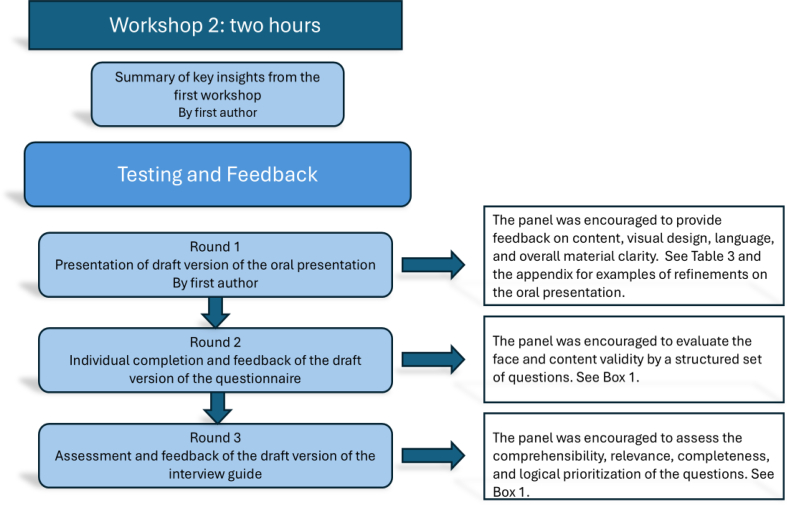
Activities of workshop two.

The second workshop (see Fig. 3) was opened with a summary of key insights from the first workshop, followed by a presentation of the draft version of the oral presentation presented by the first author. The panel was encouraged to note their feedback throughout the presentation. Comments on the content, visual design, language, and overall material clarity were invited. After the presentation, participants shared their reflections in a plenary discussion.

Participants were asked to complete a draft version of the questionnaire to evaluate its face and content validity. To guide their feedback, participants were presented with a structured set of questions (see Box 1) addressing aspects such as clarity of wording, adequate response options, and any items perceived as redundant. Completed questionnaires were collected for subsequent refinement.

Box 1 **Guiding questions for assessment of evaluation tools.**

The questions in the two columns were given to the workshop panel to help them evaluate the draft versions of the questionnaire and the interview guide.

**Table:** 

Questions for evaluation of the questionnaire	Questions for evaluation of the interview guide
• Are there any questions that are difficult to understand or hard to answer? • Are the response options sufficient for your needs? • Are there any obviously missing questions in the questionnaire? • Are there any questions that feel redundant? • Is there anything about the general structure of the questionnaire that causes confusion or irritation?	• Are there any questions that are difficult to understand or hard to answer? • Are the questions prioritized properly? • Are there any obviously missing questions in the interview guide? • Are there any questions that feel redundant in the interview guide? • Are there any questions that cause irritation?

Finally, the draft interview guide was introduced. Participants were asked to assess the comprehensibility, relevance, completeness, and logical prioritization of the questions. This discussion was likewise guided by questions to ensure systematic feedback across key dimensions (see Box 1).

### 2.10 Refinement

Following the second workshop, the project lead refined the presentation based on feedback from the workshop panel. The new presentation structure drew inspiration from Ross Fisher's P-Cubed concept [43] and was substantially tailored to the target audience.

A professor of phenomenology at the University of Southern Denmark was consulted to help refine the interview guide, with particular emphasis on a lifeworld perspective [44], [45].

### 2.11 Pilot – retesting

The questionnaires were tested in a real-world setting alongside a presentation. To strengthen face validity, a digital designer later improved the physical layout of the questionnaires. The presentation and questionnaires were pilot tested for a second time during the project's first formal session.

The interview guide was tested through two semi-structured individual interviews conducted by the first author, each lasting one hour.

### 2.12 Evaluation

The oral presentation will be evaluated through future research. Older adults across 40 local settings in the Region of Southern Denmark and the Central Denmark Region will be invited to the presentation: *“Quality of Life at the End of Life: How to Prepare”*. Participants will be invited to complete a questionnaire immediately before, immediately after, and three months following the presentation to assess whether the presentation has increased knowledge and/or motivated reflections or actions related to ACP.

The interview guide developed in the present study will be used to explore factors, including information, that influence older adults' reflections and actions regarding preparation for the end of life.

### 2.13 Data collection and analysis

The data material created in the first workshop consisted of audio recordings and post-its placed on posters by all three groups. The data were used to create a prototype of the presentation, questionnaires, and an interview guide, which were presented at the second workshop.

Participants engaged in preliminary analysis during the first workshop, without explicitly referencing the analytical framework. The process was inspired by principles from the KJ Method [46], a visual and participatory approach in which each participant's individual post-it notes are collectively grouped based on perceived thematic similarities, forming ‘affinity groups’ [47] (see Diagram 1 in Appendix 2). After the workshop, the preliminary affinity diagrams from each group informed the subsequent analysis and were converted and further analyzed by the first author employing an inductive content analysis [48], [49]. To enhance the trustworthiness of the analysis, the resulting themes were discussed with the last author. The last and second author participated in the workshops, and the presentation (informed by the analysis) was subsequently delivered to the workshop members twice, providing opportunities for further feedback and refinement.

Data from the second workshop consisted of oral and written feedback. Coding categories were developed from the written data through an iterative process of open coding and abstraction in addition to inductive content analysis [48], [49].

## 3 Results

### 3.1 Findings from workshops one – identifying relevant topics and presentation approaches

The first workshop focused on refining the oral presentation titled “Quality of Life at the End of Life: How to Prepare” through three rounds: content-related themes (round 1), the design of opening and closing elements (round 2), and evaluative elements (round 3).

### 3.2 Content-related themes (round 1)

Participants identified five overarching themes central to the presentation's content (see Table 2).

**Table 2:** Content analysis of inputs.

Theme	Description	Illustrative Participant Input from Post-It Notes
(Round 1) Content-Related Themes		
Legal and Ethical Considerations	Advance directives, right to refuse treatment, and ethical dilemmas	“Ethical perspectives on end-of-life issues”, “The role of relatives when the older adult has dementia”, “Advance directive”, “Power of attorney”, “The Danish 60+ provision allowing resuscitation refusal”, “Relatives may wish for more treatment than the patient, but it is the patient's right to refuse treatment”
EOL Care Options and Pathways	Terminal care, palliative options, dying at home	“Where palliative care can be accessed”, “What to expect from different treatment options.”, “Available options for those who wish to die at home.”
Psychosocial aspects of Quality of Life	Social contact, nature, shared reflection, helplines	“It is important to seek joy. For example, through communal singing, social interaction, and meaningful experiences in nature.”, “Networks of like-minded individuals.”, “Valuable to have conversations with close family about the final stage of life.”
Communication with Healthcare Professionals	Role of professionals, open discussion of prognosis and treatment limitations	“The importance of receiving an honest answer about one's estimated prognosis.”, “There is a need for knowledge about the effects of resuscitation attempts.”
Conceptual Clarification	Confusion in public discourse about the terms of terminal care and euthanasia	“Palliative care is not the same as assisted dying.”, “Palliative care is legal and humane, and may sometimes hasten death”, “Patients are allowed to refuse treatment. It is not euthanasia.”
(Round 2) Design of Opening and Closing Elements		
Personal Resonance and Reflection	Personal approaches, use of storytelling, songs, and reflection prompts	“Easy to understand”, “Manageable and well-structured”, “Always centered on the personal perspective”, “Emphasizing that it is a difficult but important topic”, “Who matters to you, and how do you support one another?”, “Song: Be grateful for every day you have”, “Footprints – what footprints have you left behind?”, “Personal perspective – make it relatable and emotionally engaging for the audience”.
Follow-up and Network	Summary of key points, printed handout, opportunity for asking questions, and networks to share experiences	“Where can I go if I have questions later on?”, “Handout with the key points”, “Summary of the main takeaways”
(Round 3) Evaluative Elements	Perceives outcomes: new knowledge, relevance, prompted reflection, or inspired to action, constructive feedback.	“Did you gain clarity or feel more confused?”, “What are you taking with you from today?”, Was it relevant?, “Did you learn something new?”, “Is there anything you plan to do as a result?” “What were the three most important topics for you?”

The right column presents examples of inputs written by the workshop panel on post-it notes during the first workshop. The middle column provides summaries and interpretations of these inputs, while the left column displays the overarching themes used to categorize the participants' contributions.

#### 3.2.1 Legal and ethical considerations

Participants emphasized the importance of addressing legal rights and ethical dilemmas related to EOL decision-making. This included information on legal instruments such as advance directives, power of attorney, and the legal right to refuse treatment. Ethical dilemmas concerning life-prolonging treatment at the EOL were considered essential for fostering critical reflection on whether such interventions are always consistent with the patient's values and best interests.

#### 3.2.2 EOL care options and pathways

The workshop panel suggested concrete, practical information about healthcare services and treatment options at the EOL. They also highlighted the implications of receiving a terminal diagnosis, support options for dying at home, and the respective roles of general practitioners, medical specialists, and community nurses as important areas of interest, alongside the availability of private care services and criteria for hospice admission.

#### 3.2.3 Psychosocial dimensions of quality of life

Beyond medical concerns, participants underscored the significance of psychological and social well-being. Access to psychological counselling, peer support networks, cancer helplines, and opportunities for meaningful social interaction were vital. Activities such as spending time in nature, listening to music, and being with loved ones were perceived as essential for maintaining quality of life in the final phase.

#### 3.2.4 Communication with healthcare professionals

Several participants stressed the importance of open and honest conversations about prognosis and the potential benefits and limitations of medical treatment. Transparent communication about interventions like resuscitation and support in weighing the consequences of various treatment choices was considered crucial.

#### 3.2.5 Conceptual clarification

Participants identified a need to clearly distinguish between commonly misunderstood terms such as palliative care, terminal care, and euthanasia. These concepts are often confused in public discourse. Clarifying these concepts during the presentation was considered essential to support navigating the public debate and increasing awareness of the wide range of palliative care options available within the healthcare system.

### 3.3 Design of opening and closing elements (round 2)

Participants emphasized the importance of fostering personal reflection. Opinions varied on how to begin: some favored a direct approach, others preferred emotional or narrative openings. Storytelling, literature, and music were suggested. To close, a summary, printed handout, and reflective prompts were proposed. The potential for the presentation to spark further conversations and peer support networks was highlighted.

### 3.4 Evaluative elements (round 3)

Participants agreed on evaluating perceived outcomes such as knowledge gain, relevance, and key takeaways. They emphasized the importance of assessing whether the presentation prompted reflection or action. Suggestions for improvement, identification of unclear or missing content, and feedback for future development were also considered essential.

### 3.5 Findings from workshops two – testing and feedback

Feedback on the oral presentation (intervention)

Overall, the lay panel members plenary expressed high satisfaction, stating that the presentation was informative and meaningful. The healthcare professionals provided the most extensive written feedback. The analysis highlighted an ongoing balance between the complexity of the content and the importance of maintaining accessibility for older adults as the target audience (see Table 3).

**Table 3:**
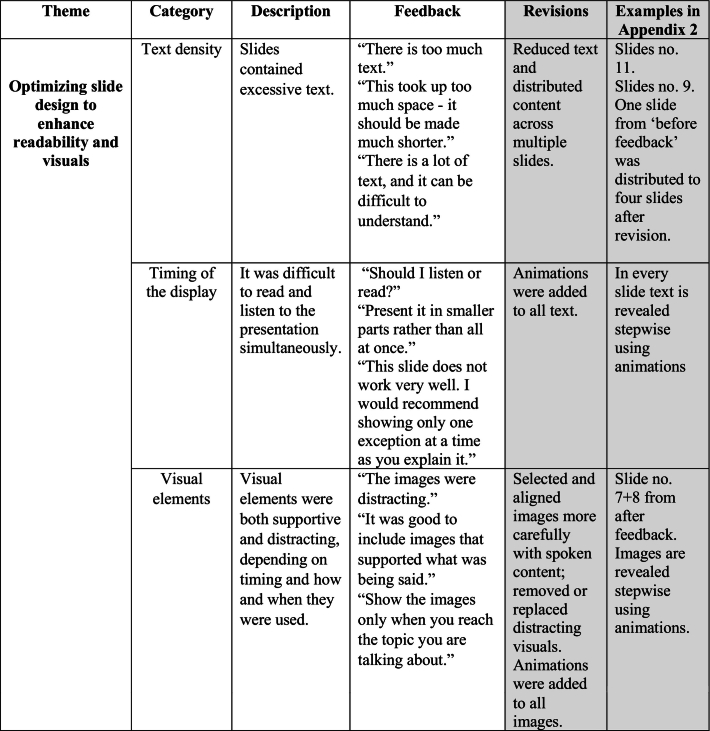

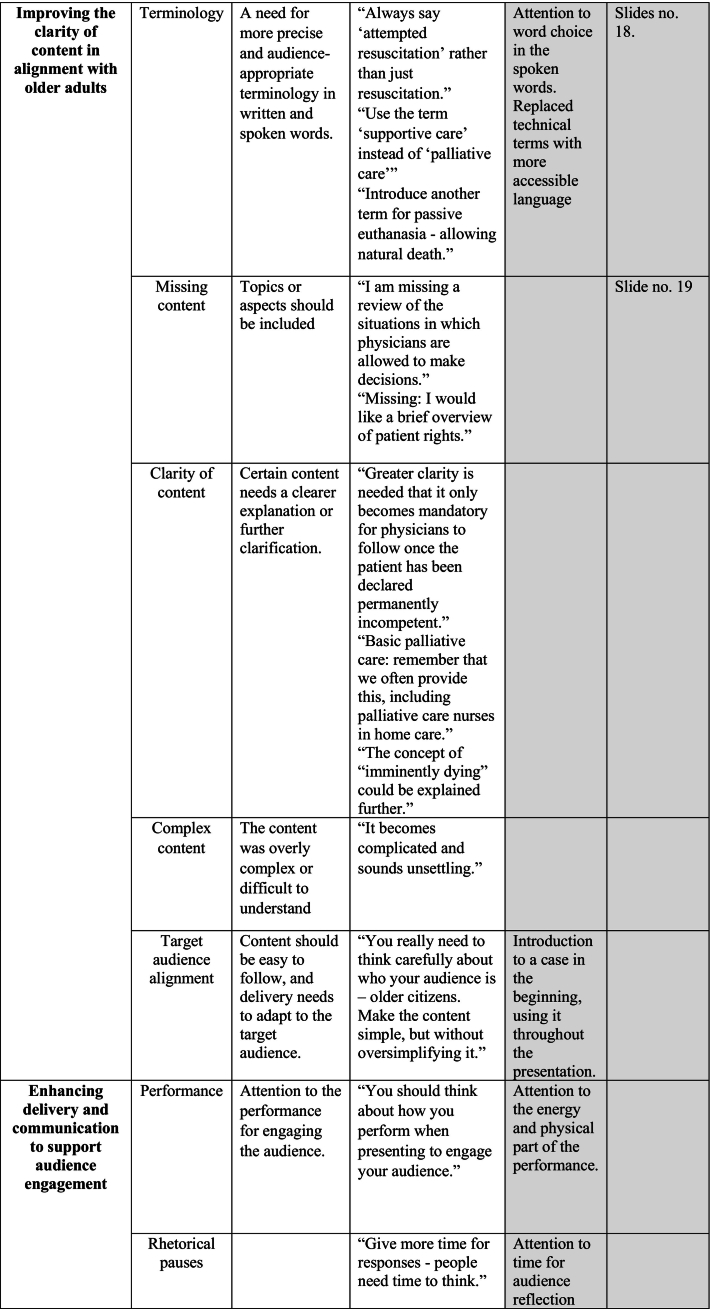
Conventional content analysis of written feedback.

The white columns show the content analysis of the feedback. The grey columns outline the revisions made to the presentation in response to the content analysis presented in the white columns and provide examples of these revisions, as shown in Appendix 2.

#### 3.5.1 Optimizing slide design to enhance readability

Feedback highlighted slide design as a central area for improvement, particularly regarding text density, timing of content display, and the use of visual elements. Feedback emphasized difficulties in processing written and spoken information simultaneously, pointing to the need to reduce text and present content in smaller segments. Visual elements were perceived as both supportive and distracting, depending on their alignment with the spoken content.

#### 3.5.2 Improving the clarity of content in alignment with older adults

Several challenges related to the content's clarity and accessibility, particularly regarding terminology, level of detail, and alignment with the target audience, were addressed. The feedback highlighted the need for clear and audience-appropriate explanations and terminology regarding medical and ethical concepts. Patient rights and decision-making processes were identified as missing content.

#### 3.5.3 Enhancing delivery and communication to support audience engagement

Feedback on presentation delivery underscored the importance of communication style and performance in supporting audience engagement, emphasizing interaction and the use of rhetorical pauses to facilitate reflection.

The PowerPoint slides were streamlined and made more visually engaging, and the overall structure was reworked to follow a narrative, story-driven approach. The content was refined to convey a single, clear message: the importance of reflecting on treatment preferences in advance and sharing them with others (see Table 3 and Appendix 2 for slide revisions).

#### 3.5.4 Feedback on evaluation tools

Feedback on the questionnaires led to a few specific revisions. Some items were identified as overly complex, often containing multiple questions in one, which made them difficult to interpret and answer. These were reformulated to ensure clarity and single-question focus (see Example of questionnaire revision in Appendix 1).

Almost no feedback was offered on the interview guide. This may be attributed to the fact that the post-presentation discussion evolved into a highly engaging and dynamic dialogue among participants, taking precedence over a detailed review of the guide.

### 3.6 Pilot – restesting

After being tested in Workshop Two, the oral presentation was revised based on panel feedback (see Table 3 and Appendix 2 for examples of slide revisions). After revising the presentation, it was recorded and emailed to the workshop panel for feedback. No further revisions were made. The last author pilot-tested the questionnaires in a real-world setting with older adults during an oral presentation on end-of-life care. In addition, key terms such as *relative* and *physician* were further defined to avoid ambiguity and ensure consistency in respondents' interpretations. Following the pilot-test of the oral presentation in a real-world setting, additional content on assisted dying was included in response to audience interest and questions.

After consultation with a professor in phenomenology, the interview guide was revised to include questions emphasizing a lifeworld perspective and decision-making processes (see the appendix: Interview guide *before* consulting a qualitative expert and Interview guide *after* consulting a qualitative expert). After the pilot-tests, no further revisions were made.

## 4 Discussion and conclusion

### 4.1 Discussion

#### 4.1.1 Summary of findings from workshop one

The findings from the workshops highlighted the importance of providing clear information about legal rights, ethical considerations, end-of-life care options, and psychosocial support. Participants stressed the need for clarity in key concepts and advocated for normalizing conversations about end-of-life issues in daily life. They emphasized the importance of the oral presentation being personally resonant, encouraging reflection, providing opportunities to share experiences, and effectively summarizing key points.

Participants emphasized the importance of including information about legal rights related to ACP and EOL care. Previous research has identified knowledge gaps regarding ACP and healthcare proxies among the general population [50], as well as limited knowledge of advance directives across populations [51]. A national Danish survey indicates that over half of clinicians specialized in chronic kidney disease had minimal or no knowledge of the national legal framework for ACP [52], suggesting a potential similar barrier in the general public. These studies support the panel members' call for information on legal aspects of ACP and EOL care, highlighting a need for improved education to enhance literacy and empower individuals to make informed decisions about their care preferences.

In addition to medical concerns, participants emphasized the importance of psychological and social well-being as essential components of quality of life in the EOL. A systematic review [53] notes that while psychosocial interventions are widely used in care for patients with incurable cancer, the evidence for their efficacy in improving health-related quality of life remains limited. The review highlights the lack of a coherent definition of psychosocial intervention and emphasizes the need for greater inter-professional clarity and collaboration. In contrast, a recent systematic review and meta-analysis found that psychological interventions significantly improve quality of life across several domains for patients with early-stage cancer [54]. A review on anticipatory grief in women with cancer emphasizes early, structured psychotherapeutic interventions in advanced illness. These interventions reduced grief responses and improved coping and emotional well-being for both patients and their families [55]. These findings suggest that while more research is needed in the context of advanced illness, the potential significance of psychosocial aspects in EOL care deserves attention.

Participants highlighted the importance of clarifying concepts such as palliative care, terminal care, and euthanasia, noting that these terms are often conflated in public discourse. This concern is supported by Danish studies demonstrating substantial misunderstanding of euthanasia and its distinction from palliative care, despite widespread public support for legalization [56] [57] [58]. By clarifying these concepts, this intervention may contribute to more informed public discussions about end-of-life care.

A key area of discussion in Workshop One concerned the design of the presentation's opening and closing elements. Participants anticipated that the presentation should encourage personal and existential reflection, rather than deliver information. Participants suggested the inclusion of storytelling to foster resonance and support reflection. The effect of this suggestion is supported by a scoping review [59] that highlights how narrative formats can stimulate emotional engagement, aid comprehension, and encourage reflection in health communication. Two studies support the use of narratives as an effective communication approach in ACP. One study explored how cancer patients engaged with ACP after receiving narrative-based information about cardiopulmonary resuscitation [60], while another case study demonstrated that narrative formats can help laypeople better understand EOL care in an educational context [61].

Recent frameworks reconceptualize ACP as a broader concept of care planning that extends beyond end-of-life preparation to include ongoing care planning throughout the illness trajectory [62]. The intervention developed in this study is aimed at older adults in the life course of ‘healthy adults and/or chronic illness’ in relation to the “care planning-umbrella” [62]. The content of the intervention addresses both ‘in-the-moment decisions’ and ‘advance decisions’ in the future life course of ‘serious illness’ and ‘end of life’, emphasizing that decision-making settings can vary.

#### 4.1.2 Strengths and limitations

By using a participatory design and framework, the study ensured the facilitation of an iterative, user-informed development. Involving patients, relatives, and healthcare professionals in shaping the intervention reflects real-world needs and values, fosters a sense of ownership among participants, and increases the likelihood of meaningful and sustainable outcomes [27]. The present study is inspired by a study from the UK, which demonstrated that community-based interventions, which include presentations and interactive workshops, can encourage reflection and dialogue about EOL planning among older adults [34]. Our study aimed to take a step further by involving a workshop panel directly in developing the early ACP intervention inspired by the participatory approach [34]. The workshop panel presented diverse perspectives, but a larger sample size may have provided additional perspectives. Although the workshop panel was small, and the study was limited in scope and context, it offers a concrete example of how co-design can be applied to ACP in a way that is accessible, and sensitive to public concerns. Structured methods, including the use of guiding questions and pilot testing, were employed to support the face and content validity of the evaluation tools. While feedback on the interview guide was limited, the dynamic discussion between the members of the workshop panel reflected a high level of engagement. Despite these limitations, the study offers a transparent example of how patients, relatives, citizens, and healthcare professionals can be involved in the early stages of intervention development within a sensitive topic area.

A limitation of this study is that three participants in the workshop panel were affiliated with the last author and two of the participants had contributed to shaping the project during its initial phases. To minimize potential bias, workshop groups were formed to prevent direct interaction between the last author and the two workshop members. Importantly, the analysis revealed notable differences in themes across the workshop groups, suggesting that diverse perspectives were expressed and that prior involvement in the project did not result in homogeneity of views. Although participant-generated affinity groups informed the analysis and the resulting themes were discussed with co-authors, a limitation of this study is that the coding of data was conducted by a single author. Parts of the workshops were audio-recorded. Unfortunately, the recordings were unusable due to poor sound quality. Audio recordings could have enriched the written data.

### 4.2 Innovation

The innovation of this study lies in its methodological and contextual approach. The study applies a participatory co-development process to the design of an early advance care planning intervention, involving a workshop panel of patients, relatives, citizens, and healthcare professionals. This approach is novel within the field of advance care planning, which has traditionally focused on patients with serious illness and interventions developed from professional perspectives.

By engaging a workshop panel in the early stages of intervention design, the study translates participatory design methods into the context of preparing for end of life. This methodological and contextual innovation enhances the relevance, acceptability, and potential impact of materials intended to support timely and meaningful engagement with ACP.

### 4.3 Conclusion

By identifying themes and formats emphasized by a panel of patients, relatives, a citizen, and healthcare professionals, our findings provide a basis for developing early ACP interventions that are perceived as meaningful and accessible. The study also contributes to understanding which domains within ACP older adults consider particularly important to address and reflect on at an early stage. These insights establish a basis for future research into how the intervention shapes public knowledge, stimulates reflection, and promotes engagement with ACP.

## CRediT authorship contribution statement

**Sabrina Westergaard Jensen:** Writing – original draft, Validation, Project administration, Methodology, Investigation, Funding acquisition, Formal analysis, Data curation, Conceptualization. **Morten Rune Blichfeldt-Eckhardt:** Writing – review & editing, Supervision. **Helene Korvenius Nedergaard:** Writing – review & editing, Supervision. **Mette Aaby Smith:** Writing – review & editing, Supervision. **Hanne Irene Jensen:** Writing – review & editing, Supervision, Methodology, Conceptualization.

## Declaration of generative AI and AI-assisted technologies in the writing process

During the preparation of this work, the authors determined the content and wrote a draft manuscript. They subsequently used ChatGPT to improve the readability and language of the manuscript. After using the tool, the authors reviewed and edited the content as needed and take full responsibility for the content of the published article.

## Declaration of competing interest

The authors declare that they have no known competing financial interests or personal relationships that could have appeared to influence the work reported in this paper.
