# Supplementary material for: “How to prepare for end of life”: Co-development of a community-based advance care planning intervention through participatory design
Source: PEC Innov. 2026 Jul 10;9:100489. doi: 10.1016/j.pecinn.2026.100489 (PMC13382466; doi:10.1016/j.pecinn.2026.100489)
Supplement: Supplementary file 1 — Supplementary material 1 [file mmc1.docx]

**Appendix 1**

Theoretical Framework for the Workshops

Participatory Design (PD), grounded in action research, emphasizes mutual learning and equitable partnerships. In full-scale PD studies, participants are often involved as co-researchers throughout all phases of the research process (34). In this study, participants were engaged as co-designers in developing the intervention. Still, they were not fully involved in data analysis or dissemination, aligning with Level 3 of Patient and Public Involvement (35). This level focuses on collaboration during the formative stages of research, including identifying needs, generating ideas, and testing potential solutions in real-world settings. The iterative and adaptive approach allows continuous refinement based on participant feedback and reflection (33).

The workshops were guided by a participatory framework designed to support meaningful and structured involvement of users in developing health interventions. Central to this framework are three interrelated modes of engagement commonly used in participatory design: telling, making, and acting (33, 36). These modes offer a flexible and iterative structure for participant engagement across different stages of development. ‘Telling’-activities allow participants to share personal experiences, concerns, and values. ‘Making’ focuses on collaborative idea generation, often using creative and dialogue-based methods, while ‘Acting’ involves engaging with preliminary prototypes to assess their relevance and usability.

This framework was chosen because it facilitates iterative, user-informed development and because it aligns with the sensitive, relational nature of end-of-life care. Involving patients, relatives, and healthcare professionals in shaping the intervention reflects real-world needs and values, fosters a sense of ownership among participants, and increases the likelihood of meaningful and sustainable outcomes (26).

Example of questionnaire revision

| Question *before* feedback | Question *after* feedback |
| --- | --- |
| **26. Are you now considering completing an advance directive?** □ Yes    □ No    □ I have already created an advance directive □ I have already created an advance directive, and I am considering modifying it  **If “No”, how likely is it that you will consider completing an advance directive within the next three months?** □ Very unlikely □ Somewhat unlikely □ Likely □ Somewhat likely □ Very likely | **26. Are you now considering completing an advance directive?** □ Yes □ Maybe □ No □ Don’t know □ I have already completed an advance directive □ I have already completed an advance directive, and I am considering modifying it |

Interview guide *before* consulting a qualitative expert

(Non-validated English translation)

**Interview guide. Citizens**

**Introduction:**

- Call by prior agreement. Ask if the participant still wishes to take part.
- Ensure it’s a suitable time, lasting about 30 minutes.
- Thank you for your participation in the study.
- Brief repetition of purpose:
  - **The purpose is to gain a nuanced and deeper understanding of whether the information from the presentation has been relevant and influenced the citizen’s initiatives regarding end of life.**
- Repetition that participation is voluntary, and that it is completely acceptable to withdraw from the study without explanation both during and after the interview (until the data have been analyzed).
- All responses will be treated in pseudonymized form (*anonymous to everyone except the project leader).
- Any questions before the interview?
- The conversation will be recorded. Is it okay to start the recording?

| Introduction | What made you attend the presentation?  Did you have prior knowledge about topics related to the final stage of life before attending the presentation?  How did you experience the presentation?   - Why? |
| --- | --- |
| Relevance, new knowledge | How was the presentation relevant to you?  Did you gain new knowledge from the presentation?   - Which?   What was particularly meaningful to you? |
| Initiatives | Has the presentation led you to take any initiative regarding end of life?   - Which? (e.g., talked with relatives, advance directive)   How do these initiatives make sense to you?  Have you discussed the content of the presentation with family or friends afterward?   - What reactions or conversations arose from this?   How do you view planning your own end of life now, compared to before the presentation? |
| Closing | I have no further questions. Is there anything I have not asked about that you think is important to include?  Do you have feedback on how the presentation could be improved? |

**Closing:**

- Do you have any comments or questions before we finish?
- Thank you very much for participating in the interview.

Interview guide *after* consulting a qualitative expert

(Non-validated English translation)

**Interview guide. Citizens**

**Introduction:**

- Call by prior agreement. Ask if the participant still wishes to take part.
- Make sure it’s a convenient time, lasting about 60 (+/-) minutes.
- Thank you for your participation in the study.
- Brief repetition of purpose:
  - **We would like to understand what matters to you when making decisions about end of life, and what role the information from the presentation has played in your reflections and any subsequent actions.**
- Repetition that participation is voluntary, and that it is completely acceptable to withdraw from the study without explanation both during and after the interview (until the data have been analyzed).
- All responses will be treated in pseudonymized form (*anonymous to everyone except the project leader).
- Any questions before the interview?
- The conversation will be recorded. Is it okay to start the recording?

| Introduction  Context - lifeworld | How would you describe your daily life right now?   - In terms of work, activities, family, friends, etc. - Do your days/weeks typically look like this?   What is particularly important to you in your everyday life? |
| --- | --- |
| Transition – motivation for participation | Do you often attend lectures or talks?  What made you attend the presentation about preparing for the final stage of life?   - For example, experiences with illness, death, planning?   Were you already familiar with some of the topics?  How did you feel about talking about end of life before attending the presentation? |
| During the presentation – the experience itself | Could you describe what it was like to attend the presentation about the end of life?   - How did you experience the atmosphere and the way the information was presented?   Is there anything from the presentation that you remember particularly clearly?   - Something that moved you, surprised you, or made you reflect?   What was important for you to gain knowledge about? |
| Decision-making processes – influencing factors | Have you already made any decisions regarding your final stage of life?   - If not, why?   When you need to make decisions in your life, what do you need to make them?   - Who or what influences your decisions?   What might make it difficult for you to make decisions about the final stage of life?  Do concerns, hopes, or fears influence your decisions about what should happen at the end of life?  How do you feel about the possibility that your reflections and decisions may change over time? |
| After the presentation - resonance | Have you had any new thoughts about the end of life after the presentation?   - If yes, was it due to something that was said during the presentation?   Has the presentation made you reflect on what creates value in your everyday life? |
| Actions and conversations | Have you talked to anyone about the end of life after attending the presentation?   - If yes, how did it come about? - How was it for you to have that conversation? |
| Feedback | Do you have any feedback on how I could improve the presentation? |
| Closing | How has it been for you to talk with me about these topics today?  I have no further questions. Is there anything I have not asked about that you think is important to include?  Thank you very much for participating in the interview |
