# Supplementary material for: “How to prepare for end of life”: Co-development of a community-based advance care planning intervention through participatory design
Source: PEC Innov. 2026 Jul 10;9:100489. doi: 10.1016/j.pecinn.2026.100489 (PMC13382466; doi:10.1016/j.pecinn.2026.100489)
Supplement: Supplementary file 2 — Supplementary material 2 [file mmc2.docx]

**Appendix 2**

Script for Workshop One (in Danish)

Drejebog til Workshop One: Input til oplæg og spørgeskema+interviewguide

Tid: 02 SEP 2024 kl 15.30-18.30

Sted: Overhuset

Deltagere: 13 personer

Facilitator: Sabrina Westergaard Jensen

| Tid | Punkt | Indhold | Form og faciliteringsnoter | Materialer |
| --- | --- | --- | --- | --- |
| 15.40 | **Indflyvning og program** | **Velkommen v Sabrina Westergaard Jensen**   - Personlig præsentation - Praktik: Samtykkeerklæring, kørselsseddel, toiletter.   **Agenda**   - Baggrund, præsentationsrunde, aktivitet 1, mad, aktivitet 2, afslutning. | Plenum  To slides | - Samtykkeerklæring - Kørselssedler |
| 15.45 | **Baggrund for projektet** | **Sabrina Westergaard Jensen præsenterer** | Plenum  Tre slides |  |
| 15:50 | **Præsentationsrunde** | Sabrina starter | Plenum  Èt slide |  |
| 16.00  16.00  16.05  16.20 | **Aktivitet 1: Ideer til oplæg**  **Runde 1: Think**  **Runde 2: Pair**  **Runde 3: Shair** | **Introduktion til aktivitet 1**  **Del 1: Emner til indhold/Vigtigste emner**   - Hvordan de 3 runder kommer til at forløbe - Forberedelsestanker - Deltagere skriver egne noter på post-it’s - Inputs diskuteres i hver enkelt gruppe   **Affinity diagram**   - Clustering post-its   Moderatorer deler gruppens inputs | Plenum  Slides  Enkeltvis  Gruppevis  Alle ideer gennemgås, clustres og gives evt. overskrifter.  Plenum  Èn gruppe lægger ud, de to andre supplerer efterfølgende. | - Post-it’s - Penne - A3 papir til post-it’s |
| 16.30  16.30  16.35  16.50 | **Aktivitet 1: Idéer til oplæg**  **Runde 1: Think**  **Runde 2: Pair**  **Runde 3: Shair** | **Introduktion til aktivitet 1**  **Del 2: Emner til intro/afslutning**   - Deltagere skriver egne post-it’s - Diskuterer i hver enkelt gruppe   **Affinity diagram**   - Clustering post-its   Moderatorer deler inputs | Plenum  Slides  Enkeltvis  Gruppevis  Alle ideer gennemgås, clustres og gives evt. overskrifter.  Plenum  Èn gruppe lægger ud, de to andre supplerer efterfølgende. | - Post-it’s - Penne - A3 papir til post-it’s |
| 17:00 | **Aftensmad** |  |  |  |
| 17.30  17.30  17.35  17.50 | **Aktivitet 2: Spørgsmål til evaluering af oplæg**  **Runde 1: Think**  **Runde 2: Pair**  **Runde 3: Shair** | **Introduktion til aktivitet 2**   - Hvordan de 3 runder kommer til at forløbe - Deltagere skriver egne post it’s - Diskuterer i hver enkelt gruppe   **Affinity diagram**   - Clustering post-its   Moderatorer deler inputs | Plenum  Slides  Enkeltvis  Gruppevis  Alle ideer gennemgås, clustres og gives evt. overskrifter.  Plenum  Èn gruppe lægger ud, de to andre supplerer efterfølgende. | - Post-it’s - Penne - A3 papir til post-it’s - A3 papir til post-it’s |
| 18.00 | **Tak for i dag** | Kommentarer + plan for næste workshop | Plenum, slide |  |

Illustrations of feedback-informed revisions of PowerPoint-slides referred to in Table 3

Presentation before feedback Presentation after feedback

11


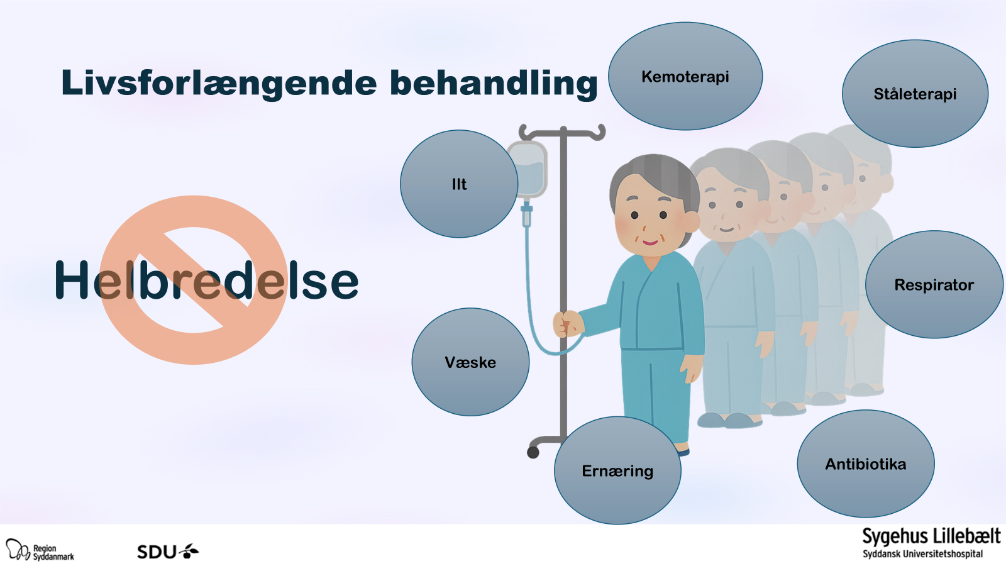

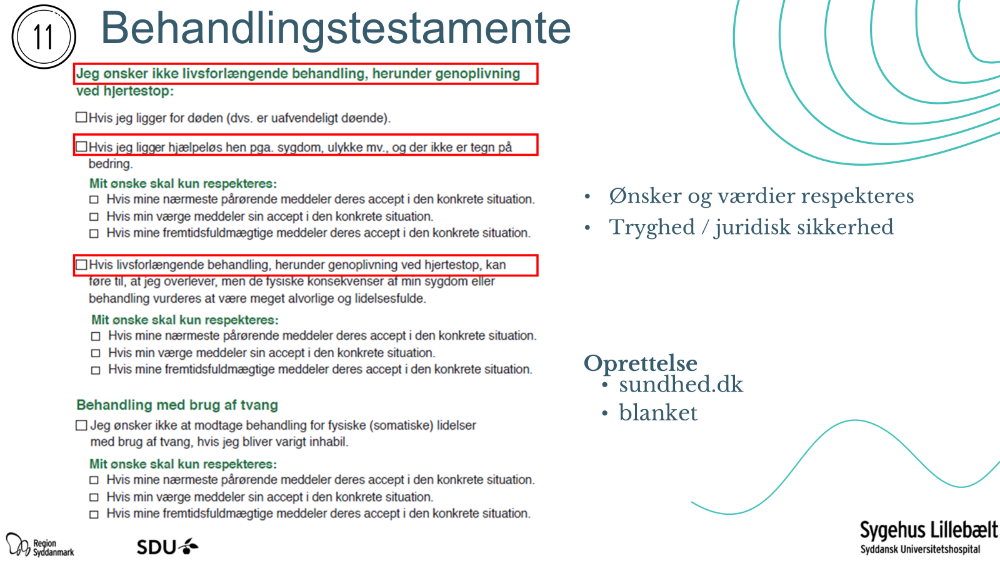

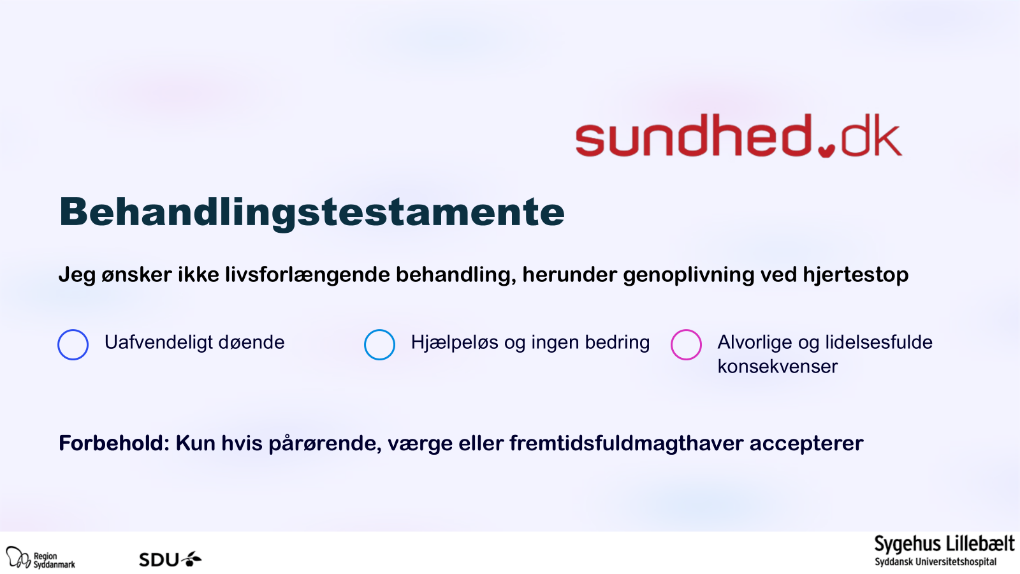

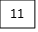


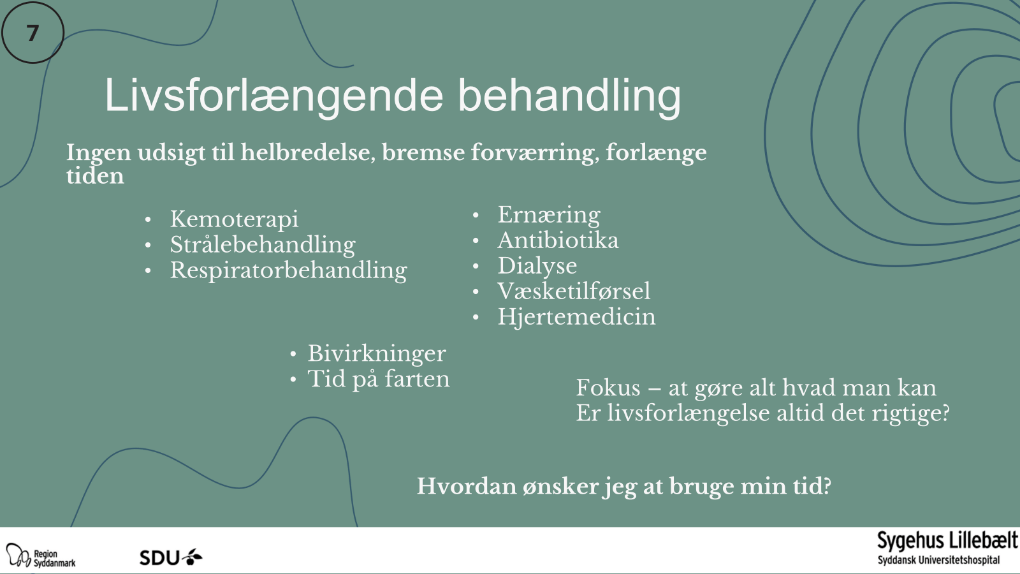


7

Deleted image


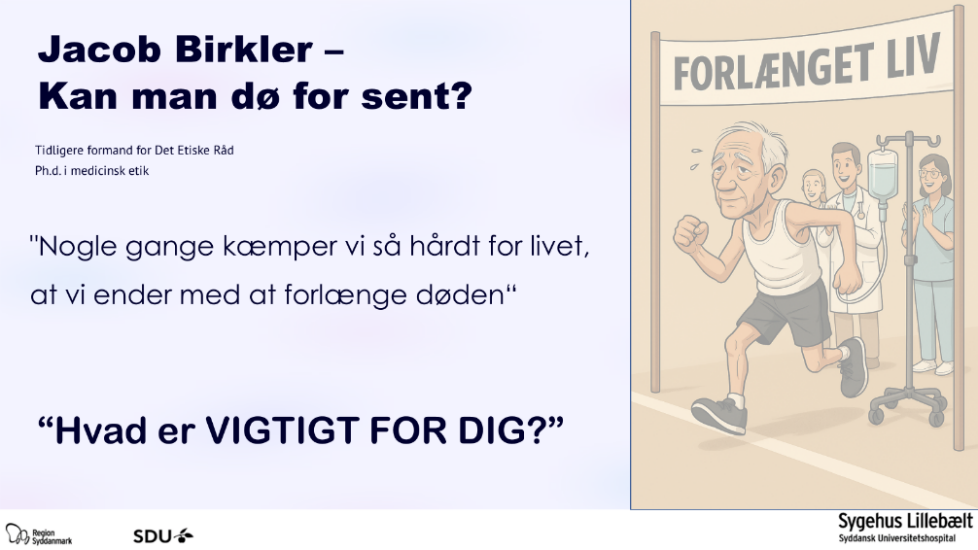

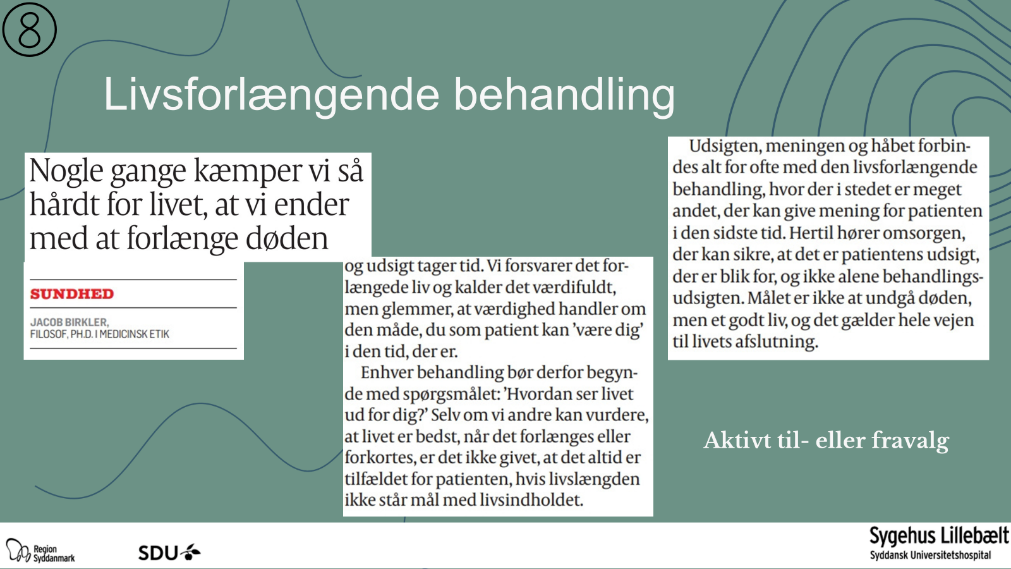


8


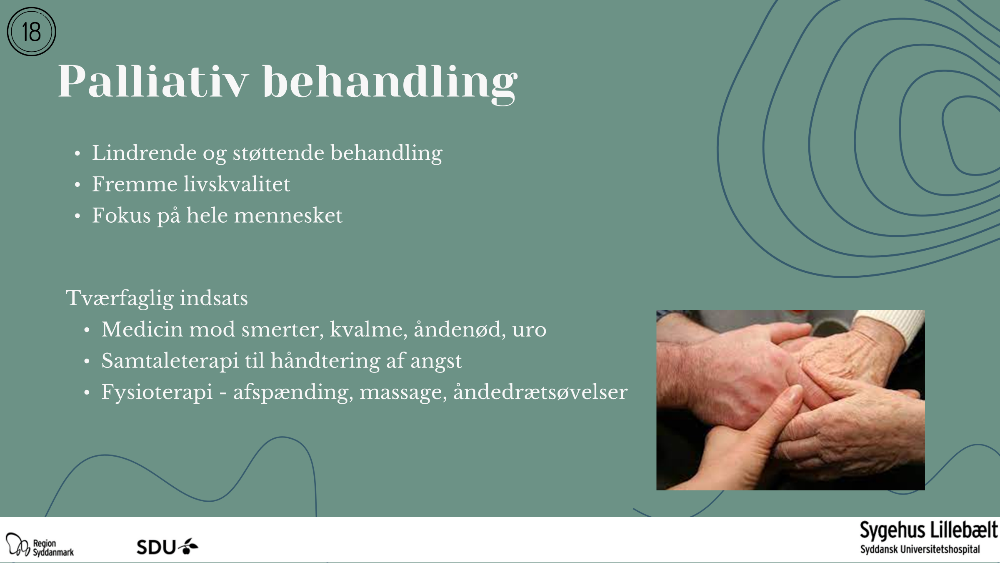

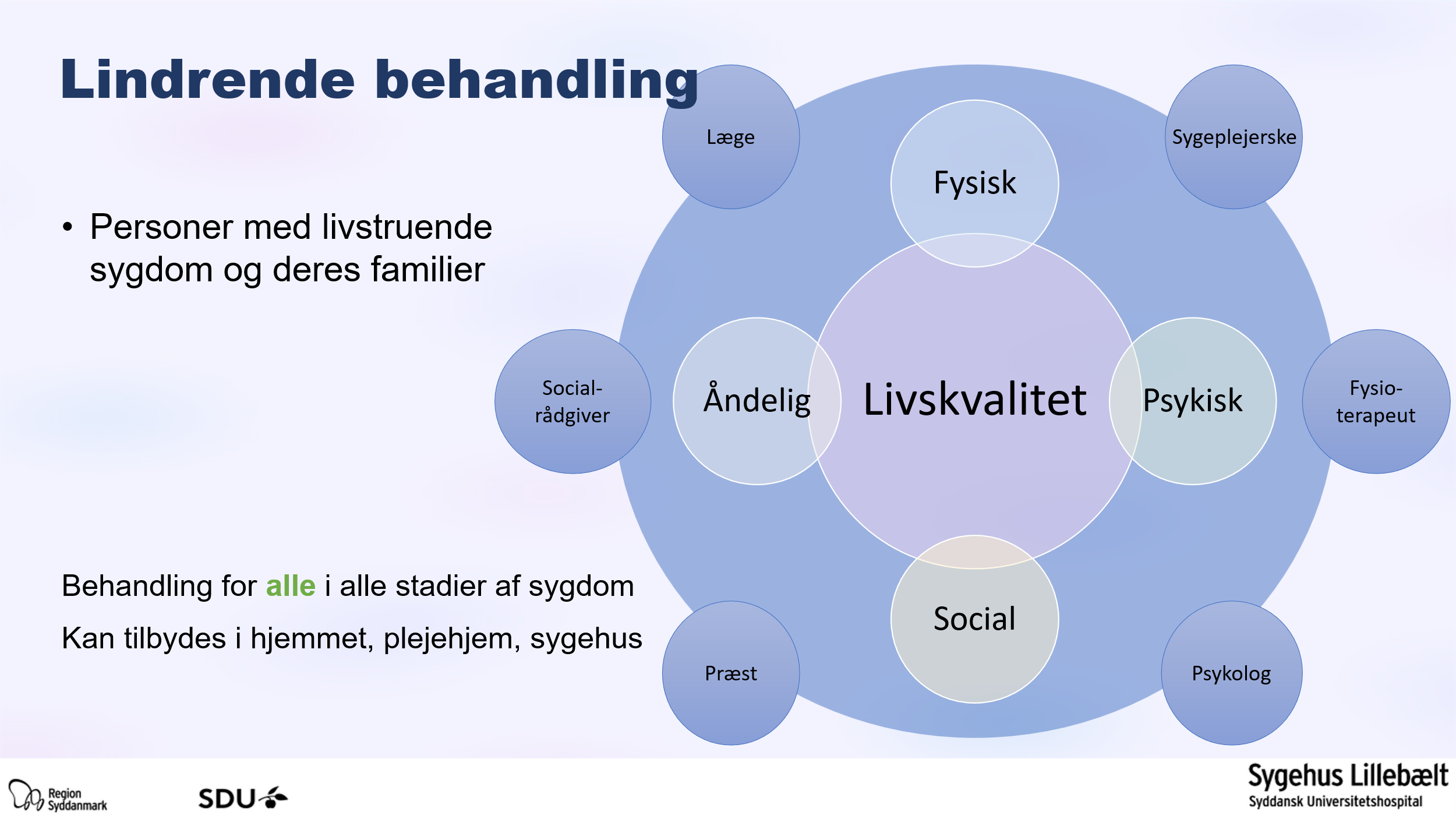


18

Deleted image


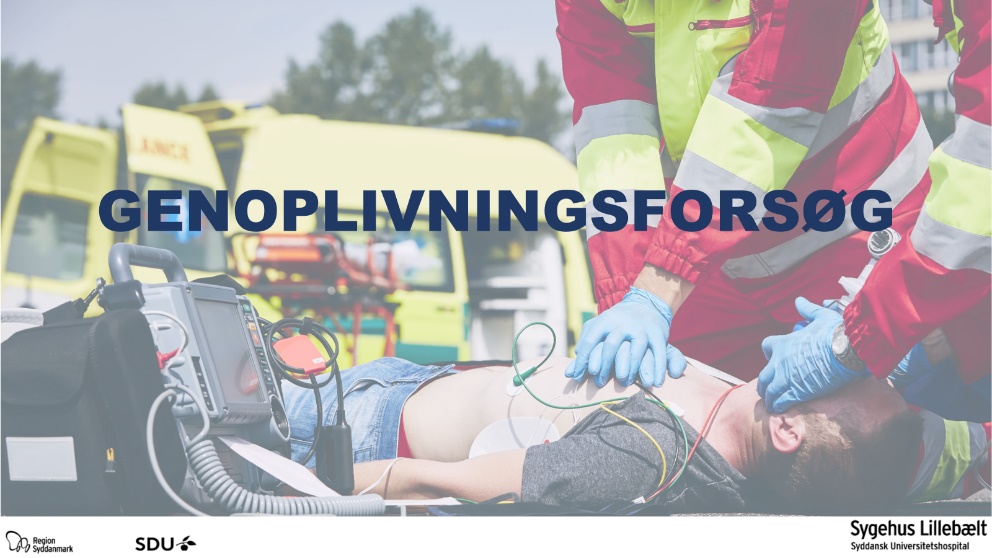

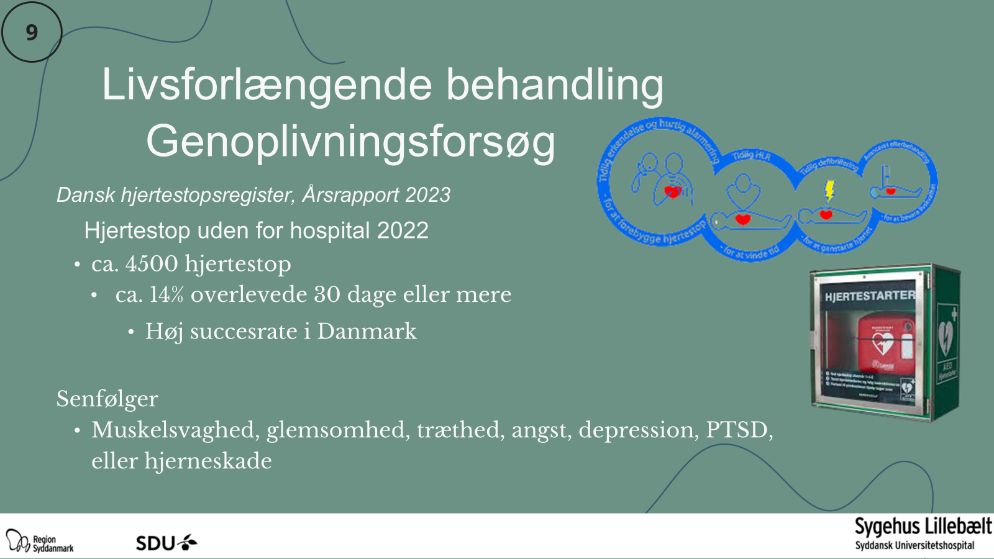


9


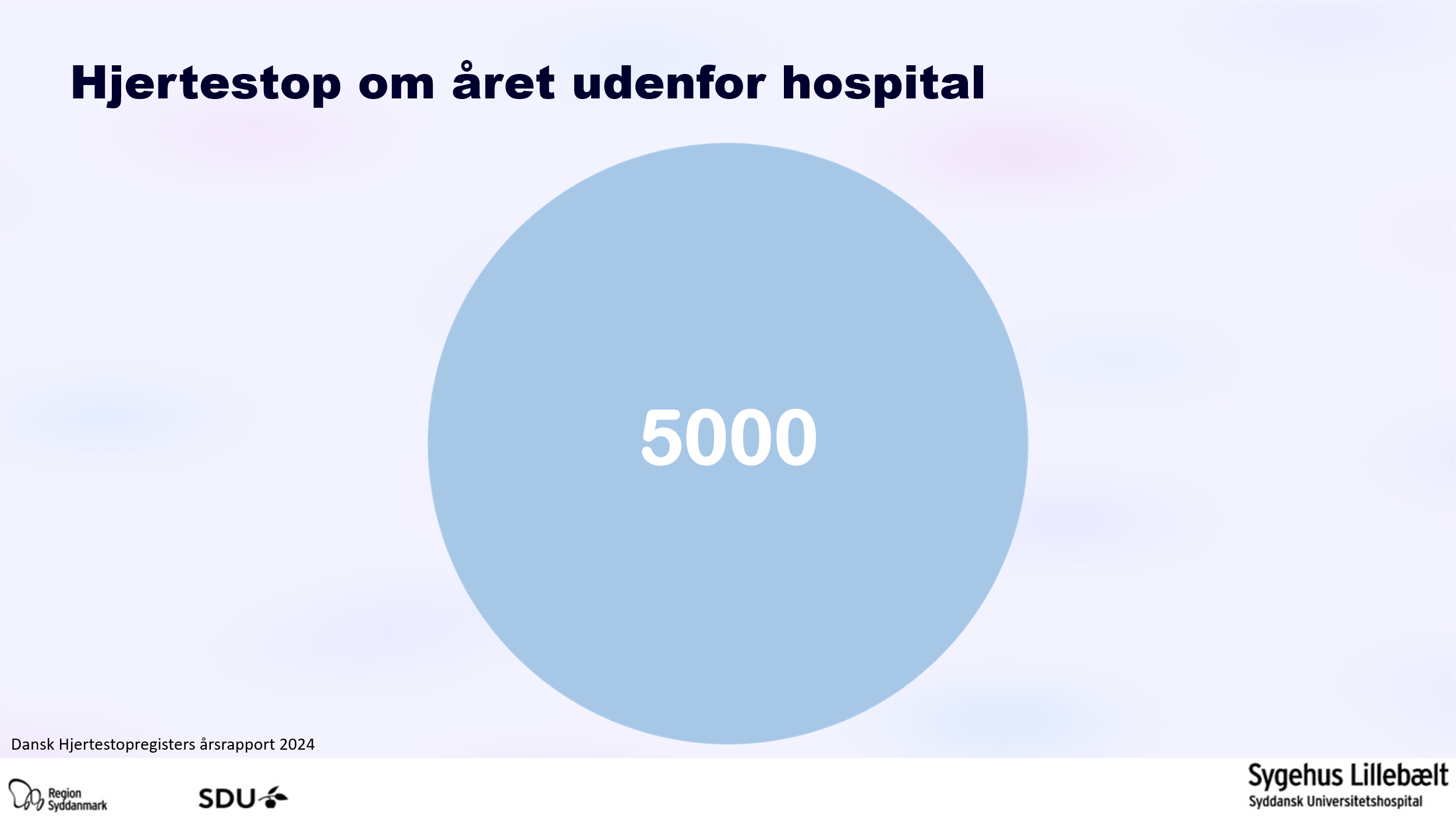


9


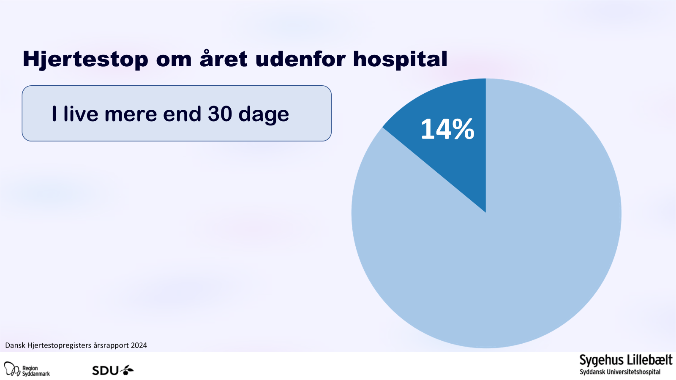


9


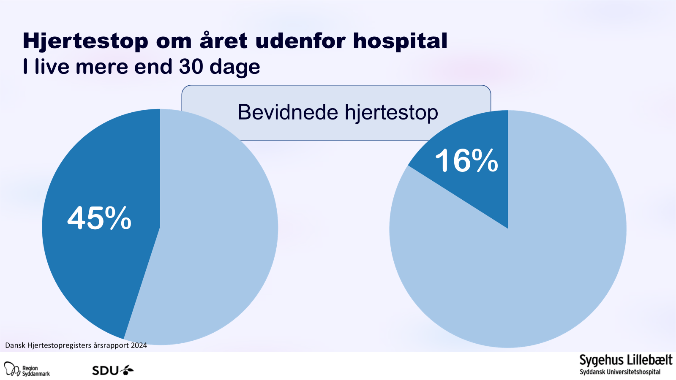


9


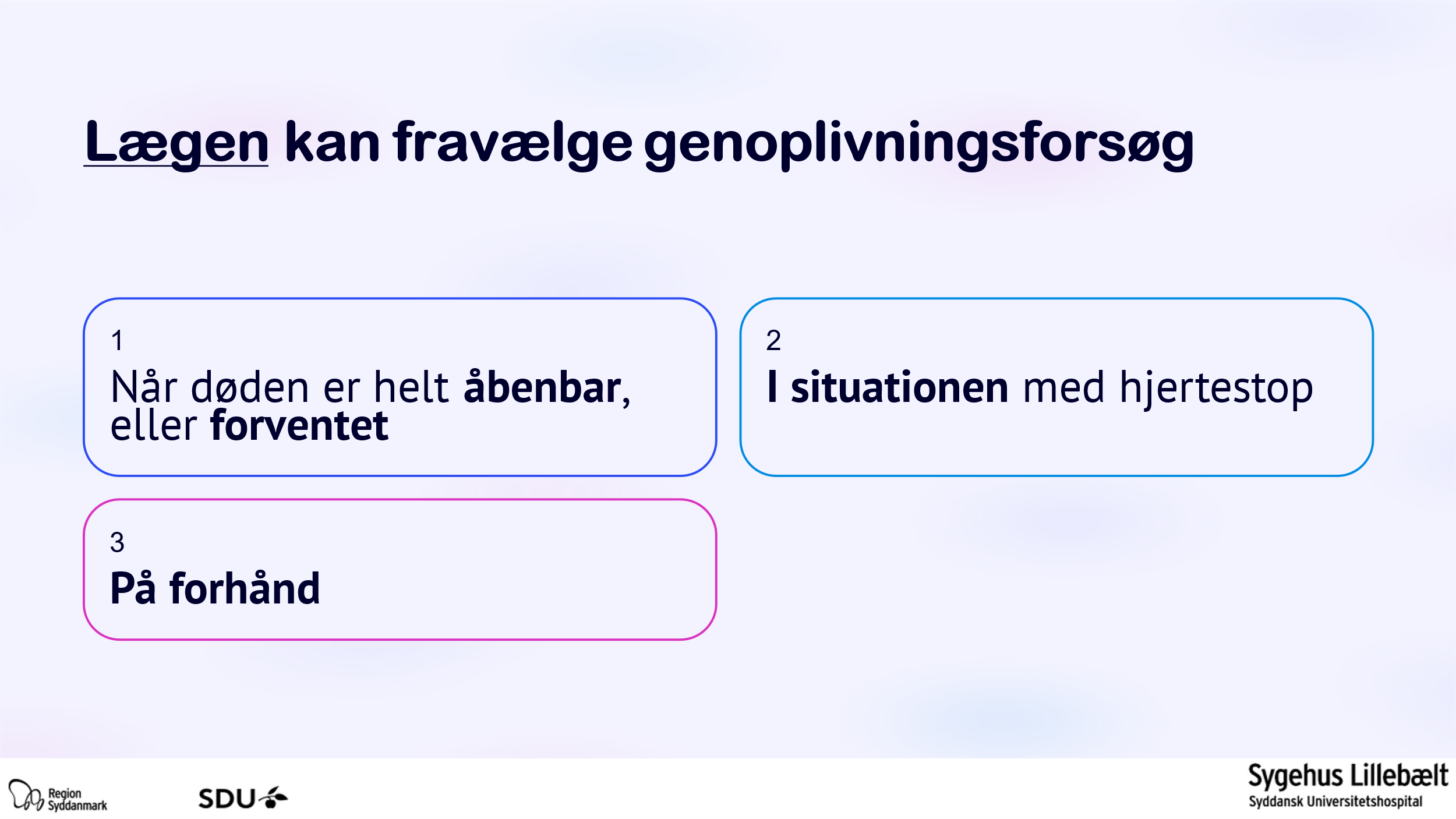


19


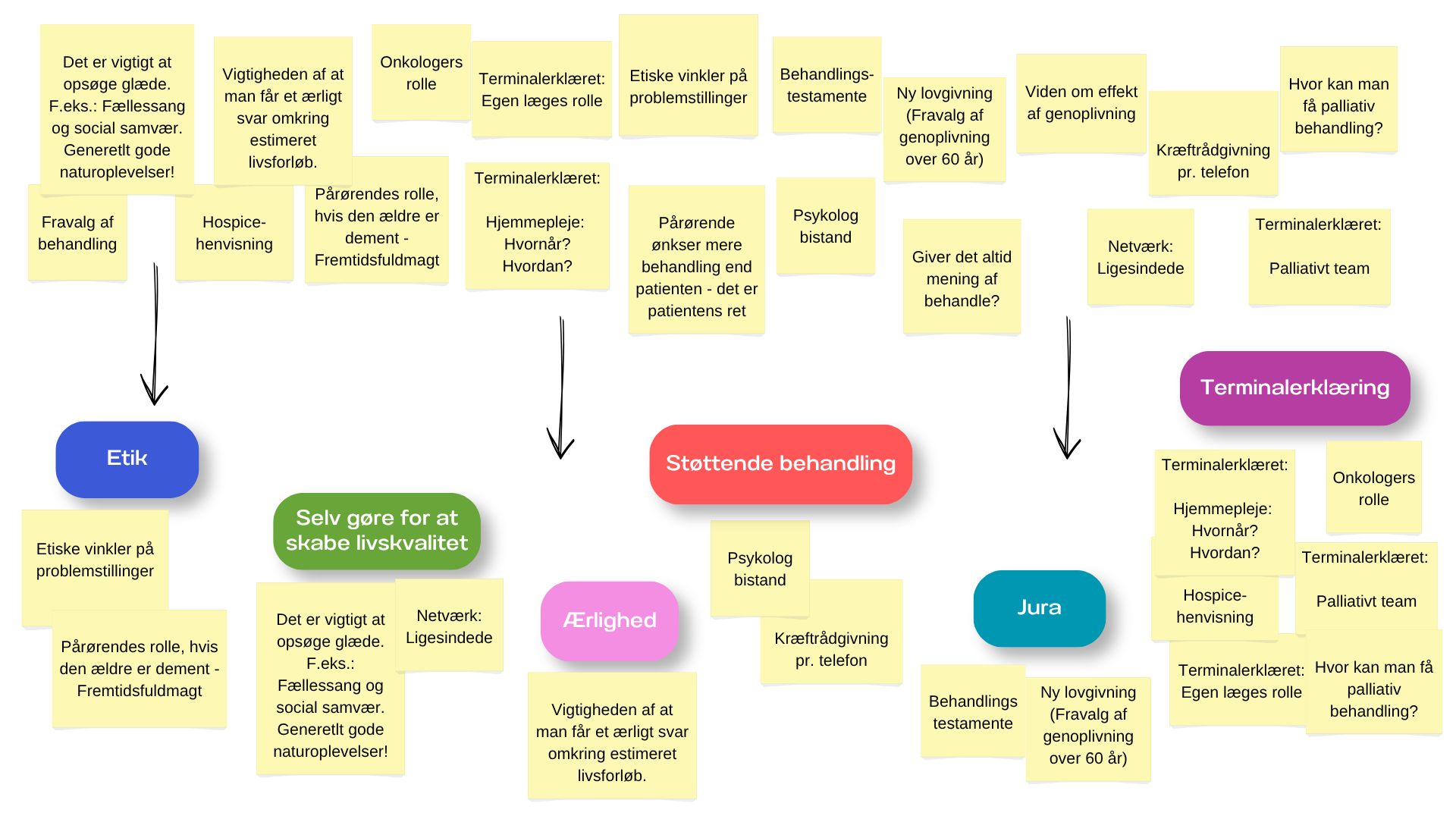
Diagram 1. Affinity diagram with post-it’s written in Danish. Workshop 1: example from Group 1, Round 1.
